# Consequences of orthodontic treatment in malocclusion patients: clinical and microbial effects in adults and children

**DOI:** 10.1186/s12903-016-0308-7

**Published:** 2016-10-28

**Authors:** Li Guo, Ying Feng, Hong-Gang Guo, Bo-Wen Liu, Yang Zhang

**Affiliations:** 1 Department of Stomatology, Ninth Hospital of Xi’an, No. 151, 2nd Ring Road East, Xi’an, 710054 People’s Republic of China; 2 Department of Orthopedic, Engineering University Hospital of PAPF, Xi’an, 710086 People’s Republic of China; 3 Department of Orthodontics, School of Stomatology, China Medical University, Shenyang, China

**Keywords:** Malocclusion, Orthodontic treatment, Fixed orthodontic appliances, Pathogens

## Abstract

**Background:**

Malocclusion is a common disease of oral and maxillofacial region. The study was aimed to investigate levels changes of periodontal pathogens in malocclusion patients before, during and after orthodontic treatments, and to confirm the difference between adults and children.

**Method:**

One hundred and eight malocclusion patients (46 adults and 62 children at the school-age) were randomly selected and received orthodontic treatment with fixed orthodontic appliances. Subgingival plaques were *Porphyromonas gingivalis (P.gingivalis), Fusobacterium nucleatum (F. nucleatum), Prevotella intermedia (P. intermedia) and Tannerella forsythensis (T. forsythensis)* collected from the observed regions before and after treatment. Clinical indexes, including plaque index (PLI), gingival index (GI), sulcus bleeding index (SBI), probing depth (PD) and attachment loss (AL) of observed teeth were examined.

**Results:**

The detection rates of *P.gingivalis*, *F. nucleatum*, *P. intermedia* and *T. forsythensis* increased from baseline to the third month without significant difference, and then returned to pretreatment levels 12 month after applying fixed orthodontic appliances. Adults’ percentage contents of *P.gingivalis*, *F. nucleatum*, *P. intermedia* and *T. forsythensis* were significantly higher than those of children at baseline and the first month, but not obvious at the third month. PLI and SBI were increased from baseline to the first and to the third month both in adults and children groups. Besides, PD were increased from baseline to first month, followed by a downward trend in the third month; however, all patients were failed to detect with AL.

**Conclusions:**

Periodontal and microbiological statuses of malocclusion patients may be influenced by fixed orthodontic appliances in both adults and children, more significant in children than in adults. Some microbiological indexes have synchronous trend with the clinical indexes. Long-term efficacy of fixed orthodontic appliances for malocclusion should be confirmed by future researches.

## Background

Malocclusion together with dental caries and periodontal disease are known as the three most common diseases in oral and maxillofacial regions [1, 2]. Malocclusion is defined as a misalignment of the teeth or an incorrect occlusion between the upper and lower dental arches [3]. Malocclusion has a significant effect on craniofacial development, oral health and function, as well as the patients’ appearance, and more serious to induce harm to systemic health of patients. Fixed orthodontic appliances is one of the most important methods applied in the course of orthodontic treatment due to its convenience and efficiency [4, 5]. Commonly used by orthodontists, fixed orthodontic appliances may result in a majority of complications at the same time, such as gingival inflammation or swelling, bleeding, hyperplasia and even slight attachment loss (AL) during treatment [6]. Besides, the stagnant zones created around fixed orthodontic appliances are in favor of the accumulation of bacterial plaques, and their colonization and reproduction [7]. Since the incidence of periodontitis increases with age [8], it is inadequate to determine that the orthodontic treatment of the juvenile period is related to the occurrence of periodontitis after middle age. With respect to the above, there is a need to clarify clinical and microbial effects of orthodontic treatment with fixed orthodontic appliances in children and adults to provide theoretical basis for the treatment of periodontal disease.

At present, researches on the relationship between fixed orthodontic appliances and periodontal disease focus more on clinical parameters on the basis of quantitative PCR Technology, instead of the effect of fixed orthodontic treatment of periodontal bacteria. There are some related subgingival periodontopathogens reported in previous studies, such as *Porphyromonas gingivalis (P.gingivalis), Fusobacterium nucleatum (F. nucleatum), Prevotella intermedia (P. intermedia)* and *Tannerella forsythensis (T. forsythensis)*, which have been suggested to possess close relationships with the development of chronic periodontitis [9–11]. Furthermore, to confirm the effect of fixed orthodontic appliances on periodontal tissue and oral microecological changes, simultaneous detection of specific pathogenic bacteria and clinical indexes is therefore needed to clarify current concerns. Oral microecological balance is indeed the basis of maintaining oral health in the human body [12]. The dynamic variation of *P.gingivalis*, *F. nucleatum*, *P. intermedia* and *T. forsythensis* and clinical examinations for periodontal tissues were therefore taken in this study to interpret basic characteristics of periodontal tissues of malocclusion patients and the difference between adults and children.

## Methods

### Study subjects

The study incorporated a total of 108 malocclusion patients (ranging from 8 to 32 years old) admitted in the Ninth Hospital of Xi’an during December 2010 to December 2013. Among them, there were 46 adults (18–32 years old) and 62 children at the school-age (8–15 years old). After recruitment, written informed consents were obtained from each participant and their guardians (mainly children) before the enrollment and the performance of the study. The diagnosis of malocclusion was in strict accordance with the criteria mentioned by Grabowski et al. [13] and Oliveira et al. [14]. In addition, following inclusion criteria were as applied: (1) patients who were confirmed to have no previous history of periodontal diseases or oral mucosal diseases; (2) patients without caries or dental fillings; (3) patients without obvious oral habits, without the habit of gum swelling or bleeding history; (4) patients who did not take antibiotics or hormones therapies one month before the experiment; (5) female patients who did not in their menstrual, pregnancy or lactation period; (6) patients had no previous history of systemic diseases or other factors that might affect bone metabolism. All patients were treated by straight wire appliance after diagnosis. The first molars were banded and a 0.5–1.0 mm diameter tube was soldered on the palatal side of the first molar band; also, the premolars were braced. The first molars and the premolars were all attached with 1.1 mm diameter stainless steel retaining wires (Reward, China).

### Experimental methods and periodontal clinical index

Both sides of mandibular central incisors and the mandibular first/second premolars were selected as the observed teeth. In addition, patients’ periodontal statuses were recorded and established at their first visit to the hospital. By using a single blind method that the clinician was unaware of the experiment status, the periodontal examination was done by the same clinician with a labeled periodontal probe (YDM, Japan). Plaque index (PLI) [15], sulcus bleeding index (SBI) [16], probing depth (PD) [17] and AL [18] of observed teeth were examined in triplicates.

Subgingival plaques were collected at medial and distal buccal axial ridge of the observed teeth at three different time points (baseline data of before orthodontic treatment, one month and three months after treatment), and stored at −70 °C for further usage. Subsequently, frozen samples were dissolved at room temperature of 37 °C, and then were concussed uniformly with a vortex oscillator, washed with Tris–HCl buffer wash once, lysozyme cleavage, followed by a conventional phenol-chloroform extraction procedure. The DNA sample were finally stored at −20 °C for real-time polymerase chain reaction (real-time PCR) experiment.

### Quantitative real-time PCR

The collected DNA samples were studied using real-time PCR according to the manufacturer’s instructions [19]. The experiment was repeated three times with similar results. PCR primers were designed by Primer Premier 5.0 software (PREMIER Biosoft International, Palo Alto, CA, USA) and were synthesized by Invitrogen Biotechnology Co., Ltd (Beijing). The positive controls of *P.gingivalis*, *F. nucleatum*, *P. intermedia* and *T. forsythensis* were the standard bacterial strains provided from the laboratory of Ninth Hospital of Xi’an. Table 1 showed primer sequences and their lengths [20–22]. The 25 μl-PCR system contained 10 × buffer (2.5 μl), 2.5 mmol/L dNTP Mixture (2.0 μl), 25 mmol/L MgCI_2_ (2.5 μl), 20 μmol/L for each forward and reverse primers (1 μl each), 5 U/μl Taq DNA polymerase (0.15 μl) (Tiangen, China), Template 2.5 μl and then made up to a final volume of 25 μl using double-distilled water. The PCR protocol on Light Cycler (LC480) (Roche Diagnostics, Mannheim, Germany) for *P.gingivalis* was: an initial denaturation step (95 °C for 5 min) and 36 cycles of denaturation (95 °C for 30 s, 60 °C for 1 min and 72 °C for 1 min) followed by annealing and extension steps (72 °C for 2 min). An initial denaturation step at 94 °C for 5 min was conducted for the remaining three indexes, followed by 30 cycles of denaturation (94 °C, 94 °C and 95 °C for 30 s for *F. nucleatum*, *P. intermedia* and *T. forsythensis*, respectively, 60 °C for 30 s and 72 °C for 1 min) and the final annealing and extension steps (72 °C for 2 min, 2 min, and 3 min, respectively).

**Table 1:** Primer sequences for *P.gingivalis*, *F. nucleatum, P. intermedia* and *T. forsythensis*

	Sequences	Amplified fragment length
*P.gingivalis*	F: 5′-AGGCAGCTTGCCATACTGCG-3′	404 bp
	R: 5′-ACTGTTAGCAACTACCGATGT-3′	
*F. nucleatum*	F: 5′-AGAGTTTGATCCTGGCTCAG-3′	408 bp
	R: 5′-GTCATCGTGCACACAGAATTGCTG-3′	
*P. intermedia*	F: 5′-CGTGGACCAAAGATTCATCGGTGGA-3′	259 bp
	R: 5′-CCGCTTTACTCCCCAACAAA-3′	
*T. forsythensis*	F: 5′-GCGTATGTAACCTGCCCGCA-3′	641 bp
	R: 5′-TGCTTCAGTGTCAGTTATACCT-3′	

Note: *P.gingivalis Porphyromonas gingivalis, F. nucleatum Fusobacterium nucleatum, P. intermedia Prevotella intermedia, T. forsythensis Tannerella forsythensis*

### Agarose gel electrophoresis

The PCR products were subsequently separated by electrophoresis in 0.3 % agarose gels. The final images were then acquired and observed in a ChemiDoc (Bio-Rad, Hercules, USA) gel documentation system.

### Statistical analysis

Data analysis was performed using SPSS software (Version 17.0; SPSS, Chicago, IL). *t* test was applied in the study to analyze differences between adults and children at the same time points, as well as the difference within the adults group and the children group at different time points. A bilateral *P* value of less than 0.05 was considered statistically significant.

## Results

### The detection results of microbial indicators

On the day following the removal of fixed orthodontic appliances, the positive percentage of the four pathogens was 40.1, 66.0, 32.0 and 48.9 %, respectively. Besides, as for the total counts of bacteria detected by real-time PCR, corresponding results were shown in Table 2. Furthermore, detection rates of the four pathogens showed stable trends among adults during three different time points. But the total detection rates in children increased overtime. Besides, as for the differences between adults and children at the same time points, the detection rates in adults exhibited obvious elevated percentages of pathogens both at baseline and after treatment than in children, but without apparent differences (all *P* > 0.05).

**Table 2:** The percentage contents and detective amount of the *P.gingivalis*, *F. nucleatum, P. intermedia* and *T. forsythensis* in adults and adolescents

	Baseline		One month after treatment		Three months after treatment	
	Percentage contents	Detective amount (log10)	Percentage contents	Detective amount (log10)	Percentage contents	Detective amount (log10)
*P.gingivalis*						
Adults	0.090 ± 0.047^*^	6.30	0.392 ± 0.621^*^	6.26	0.451 ± 0.545	6.20
Adolescents	0.005 ± 0.013	6.27	0.072 ± 0.064	6.23	0.278 ± 0.780	6.18
Total	0.025 ± 0.121		0.195 ± 0.650		0.347 ± 0.812^#^	
*F. nucleatum*						
Adults	0.083 ± 0.149^*^	6.34	0.387 ± 0.578	6.26	0.433 ± 0.479	6.11
Adolescents	0.012 ± 0.028	6.32	0.082 ± 0.066	6.26	0.317 ± 0.472	6.23
Total	0.033 ± 0.074		0.167 ± 0.356		0.401 ± 0.611^#^	
*P. intermedia*						
Adults	0.093 ± 0.148^*^	6.36	0.402 ± 0.431^*^	6.23	0.413 ± 0.512	6.15
Adolescents	0.010 ± 0.047	6.30	0.087 ± 0.079	6.20	0.255 ± 0.526	6.18
Total	0.030 ± 0.065		0.188 ± 0.518		0.349 ± 0.714^#^	
*T. forsythensis*						
Adults	0.081 ± 0.061^*^	6.30	0.394 ± 0.572^*^	6.18	0.488 ± 0.509^*^	6.08
Adolescents	0.009 ± 0.027	6.27	0.091 ± 0.107	6.15	0.284 ± 0.305	6.04
Total	0.036 ± 0.101		0.145 ± 0.416		0.373 ± 0.717^#^	

Note: ^*^, compared with the percentage of adolescents, *P* < 0.05; ^#^, compared with the percentage at baseline, *P* < 0.05

In addition, as shown in Table 2, as compared to the baseline levels, percentage contents of the four pathogens in adults and children as well as the total percentage all indicated increased tendencies one month and three months after treatment. Besides, the percentage of total sample was significantly higher three months after treatment than that of the baseline level, with statistical differences (all *P* < 0.05). Adult percentage contents were significantly higher than that of children at baseline for all pathogens, and at one month after treatment for *P.gingivalis*, *F. nucleatum*, *P. intermedia* and *T. forsythensis* (all *P* < 0.05), but there was no significant difference between adults and children in the third months after treatment except for *T. forsythensis* (all *P* > 0.05).

### The detection results of PLI, SBI, PD and AL

Adults, children, and overall PLI measurements increased with time triply. Among them, when compared to that before treatment at the baseline level, PLI was significantly higher in adults one month and three months after treatment. PLI also showed a significant increase in Children three months after treatment than those levels at baseline. Overall measurements of PLI was also increased obviously one month and three months after treatment than in that at baseline, with a more significant increase trend three months after treatment. At baseline, there were significant less PLI measured in adults than in children, but there was no significant difference in the other two time points of detection (Table 3).

**Table 3:** Detection results of clinical indicators, PLI, SBI and PD, in adults and adolescents

	Baseline	One month after treatment	Three month after treatment
PLI			
Adults	0.195 ± 0.311^a^	0.610 ± 0.512^*^	0.780 ± 0.531^*^
Adolescents	0.491 ± 0.367	0.637 ± 0.444^*^	0.899 ± 0.490^*^
Total	0.345 ± 0.292	0.680 ± 0.463^*^	0.876 ± 0.462^*^
SBI			
Adults	0.253 ± 0.412	1.123 ± 0.503^*^	1.119 ± 0.148^*^
Adolescents	0.278 ± 0.457	0.993 ± 0.492^*^	1.002 ± 0.481^*^
Total	0.283 ± 0.505	0.976 ± 0.0.484^*^	0.984 ± 0.0.496^*,#^
PD			
Adults	0.056 ± 0.122^a^	1.188 ± 0.164^*^	1.047 ± 0.148^*^
Adolescents	1.015 ± 0.101	1.190 ± 0.172^*^	1.021 ± 0.104^*^
Total	1.020 ± 0.103	1.178 ± 0.180	1.100 ± 0.0.142^*,#^

*PLI* plaque index, *SBI* sulcus bleeding index, *PD* probing depth

Note: ^*^, compared with the index level at baseline, *P* < 0.05; ^#^, compared with the index level one month after treatment, *P* < 0.05

^a^compared with the index level of the adolescents at baseline

Adults, children, and overall SBI examination were also increased with time, such level was evidently increased one month and three months after treatment than these at baseline, with statistical significance (all *P* < 0.05). But there was no significant difference of SBI in patients received treatment one month and three months later, and no obvious statistical difference was found between adults and children at three different time points, respectively (all *P* > 0.05).

Adult and children DP were increased from baseline to first month, followed by a downward trend in the third month after treatment. DP showed obvious higher levels in children treated for one month and three month than those at baseline. There was significant difference regarding PD between adults and children at baseline, but not significant at other time points after treatment. In addition, PD in all the included 108 patients were all less than or equal to 1–2 mm in the three repeated tests. Furthermore, throughout the experiment, all 108 patients were not detected with AL in the triplicate test.

## Discussion

Malocclusion is the common oral disease, with the continuous improvement of living quality, an obviously increasing trend can be observed in individuals who require for orthodontic treatment [23–25]. In-depth understanding of clinical and microbiological changes during the process of malocclusion treatment is hence crucially necessary for the treatment and further prevention of periodontal diseases, which is applicable among different age groups. Under normal circumstances, most periodontal plaque microorganisms can be maintained relatively stable due to the semi-enclosed structure of gingival sulcus, but not the same with the placement of fixed orthodontic appliance [26, 27].

In this experiment, detection rates of four different pathogens were gradually increased from baseline to one month and lately three months after treatment. Upward trends were also showed in children, significantly higher at the time point of the third month after treatment than that at baseline, whereas it was stable in 46 adults. In addition, the percentage of pathogens in the overall samples increased with time especially at the time point of the third month after treatment, gradual upward trends were found both in adults and children. We may know obviously, along with the extension of treatment time, orthodontic appliance placement will change the survival condition of subgingival microorganisms and promote the propagation of the periodontal pathogen, which in turn create favorable conditions for the onset of periodontal diseases [28]. Thus it could be illustrated out that fixed orthodontic appliance might have significant different influence in microecological environment for adults and children. Under the stimulation of orthodontic appliance and due to plaque accumulation, children’ subgingival pathogens proliferation was more obvious than that in adults.

Periodontal clinical indicators were also detected in this experiment. Mandibular central incisors and premolars were determined as the object of observation, largely due to the short clinical crown and the relatively short distance of gums after direct bonding of bracket, which in turn might be vulnerable to the interference to produce inflammation. According to the experimental results, PLI of the total samples increased gradually, same results were also detected in the adults and the children subgroups. At baseline, children’ PLI was significantly higher than that of adults, which might be correlated with the reason that adults had a generally positive attitude and habits towards oral health care [29, 30]. With the start of treatment, the differences of PLI in adults and children were not obvious, which might due to the effect of the caregivers’ oral health education, and children paid more attention to oral and dental health care [31, 32]. With the extension of treatment time, PLI was speculated to be further decreased or maintained at a stable level, which should be confirmed by further long-term investigation. Further, SBI also showed a significant difference at the first and third months than that at baseline, but no significant difference were found between adult and children subgroups as well as between different time points of the first and third month. This might indicated that inflammatory reaction might be occurred in the initial stage of orthodontic treatment [33], but then stabilized and without persistent deterioration during the following periods, more investigation should be conducted to confirm such speculation with the detection of inflammatory related parameters. Sharma NC et al. mentioned in their study that both PLI and SBI were decreased following the treatment process for children applied fixed orthodontic appliances, which was suggested to have beneficial results for the reduction of plaques and bleeding [34]. This in turn suggested the important role of clinical indexes monitoring. In addition, PD is an important indicator to measure periodontal health [35, 36]. In this experiment, PD of the lower central incisors in all patients were within the healthy range during the whole observation period (≤2 mm), also none obvious AL changes were observed over the period of observation. Although there was a marked increase of PD in the first month after treatment than that at baseline, post-treatment results showed that the average value of the third month was slightly lowered than that of the first month. In addition, comparison results indicated that between different subjects, the adults’ PD was significantly higher than that in children at baseline, but there was no significant difference one month or three months after treatment.

## Conclusions

In conclusion, fixed orthodontic appliances may have an influence in the periodontal and microbiological statuses of malocclusion patients both in adults and children. Some microbiological indexes indicate synchronous trends with clinical indexes. In addition, the effect of fixed orthodontic appliances may be more significant in children than in adults. The effect of fixed orthodontic appliances still needed further research in the long run.

## Abbreviations

AL: Attachment loss
F. nucleatum: Fusobacterium nucleatum
*P. intermedia*: *Prevotella intermedia*
*P.gingivalis*: *Porphyromonas gingivalis*
PD: Probing depth
PLI: Plaque index
real-time PCR: Real-time polymerase chain reaction
SBI: Sulcus bleeding index
*T. forsythensis*: *Tannerella forsythensis*
